# Dissecting the complex regulation of pentose utilization in *Aspergillus niger*

**DOI:** 10.1016/j.crmicr.2025.100482

**Published:** 2025-09-29

**Authors:** Mao Peng, Wiebe Wennekers, Astrid Müller, Vivian Ng, Anna Lipzen, Igor V. Grigoriev, Ronald P. de Vries

**Affiliations:** a Fungal Physiology, Westerdijk Fungal Biodiversity Institute Uppsalalaan 8, 3584 CT Utrecht, The Netherlands; b US Department of Energy Joint Genome Institute, Lawrence Berkeley National Laboratory, 1 Cyclotron Road, Berkeley, CA 94720, USA; c Department of Plant and Microbial Biology, University of California Berkeley, Koshland Hall 111, Berkeley, CA 94720, USA

**Keywords:** *Aspergillus niger*, Pentose catabolic pathway, Polysaccharide degradation, XlnR, AraR, Inducer

## Abstract

L-Arabinose and D-xylose are abundantly present in nature as components of plant cell wall polysaccharides. Fungi mainly use the Pentose Catabolic Pathway (PCP) for conversion of these sugars into central carbon metabolism. The PCP has been particularly well studied in *Aspergillus niger*, by identifying the metabolic genes as well as two transcriptional activators (XlnR and AraR) that control expression of PCP genes as well as genes encoding enzymes releasing D-xylose and L-arabinose from plant polysaccharides. While it has been demonstrated that XlnR is active during growth on D-xylose and AraR on L-arabinose, the actual inducers of these regulators have not been established.

In this study, comparative transcriptomics on all intermediate PCP compounds as well as the chiral counterparts of D-xylose and L-arabinose was performed to determine which of them results in induction of AraR and/or XlnR target genes. In addition, mutants that were blocked in each of the metabolic steps of the PCP were analyzed to determine how they affected growth on all PCP intermediates, as well as L-xylose and D-arabinose. The combined results clearly demonstrated that the inducer of AraR is L-arabitol, the first metabolic intermediate from L-arabinose, while XlnR is induced by D-xylose itself, but not by L-xylose.

## Highlights

• L-Arabitol is the monomeric inducer of AraR in *A. niger*.
• D-Xylose is the monomeric inducer of XlnR in *A. niger*.
• The induction of AraR and XlnR is chiral-specific in *A. niger*.

## Graphical abstract

**Figure:**
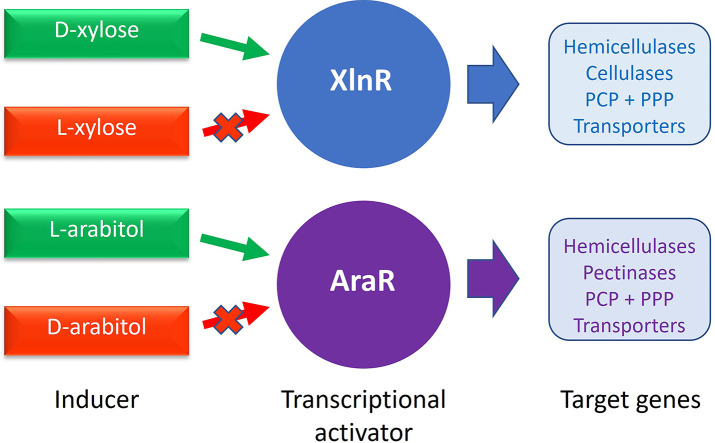

## 1 Introduction

The pentose sugars l-arabinose and d-xylose are abundantly present in nature, in particular as components of plant cell wall polysaccharides. d-Xylose is predominantly present as the backbone of the hemicellulose xylan, but is also found as a side chain of xyloglucan (another hemicellulose) and the pectin substructure xylogalacturonan (de Vries and Visser, 2001). l-Arabinose is a major component of another pectin substructure, rhamnogalacturonan I and a side chain of xylan and xyloglucan (de Vries and Visser, 2001). Due to their abundance in nature, they are important carbon sources for many microorganisms, and also have high relevance for the biobased economy. These pentose sugars have many applications and e.g., have been explored for the production of biofuels (Sharma et al., 2022) and the production of biochemicals (Das et al., 2024; Kumar et al., 2024). Most fungi convert these pentoses through the pentose catabolic pathway (PCP) (Witteveen et al., 1989), which consists of a series of reductase/dehydrogenase reactions and a final phosphorylation reaction. This final compound (D-xylulose-5-phosphate) connects the PCP to the pentose phosphate pathway (PPP) and central carbon metabolism.

The PCP has been particularly well studied in the industrially relevant ascomycete fungus *Aspergillus niger*. While initially single genes were considered for the steps of the pathway (de Groot et al., 2007; Hasper et al., 2000; Mojzita et al., 2010a, 2010b; vanKuyk et al., 2001), more recently it has been demonstrated that most steps of the pathway are catalyzed by multiple enzymes and that this apparent redundancy differs strongly between fungi (Chroumpi et al., 2021; Mueller et al., 2023). Expression of the genes from the pathway is controlled by two transcriptional activators, XlnR and AraR (Battaglia et al., 2011). XlnR also controls the expression of genes encoding xylan and xyloglucan degrading enzymes, while AraR also controls the expression of genes encoding l-arabinose and d-galactose releasing enzymes from hemicellulose and pectin (Gruben et al., 2017). AraR and XlnR are paralogs and bind to similar binding sites in the promoters of their target genes (Ishikawa et al., 2018). Interestingly, while XlnR orthologs are found in most filamentous ascomycetes, AraR orthologs are only found in Eurotiomycete fungi (Benocci et al., 2017). However, Sordariomycete and Leotiomycete fungi have a functionally similar regulator (ARA1) which has no significant sequence similarity with AraR from *A. niger* (Benocci et al., 2017).

It has been reported that the presence of d-xylose results in activation of XlnR (van Peij et al., 1998a), while the presence of l-arabinose results in activation of AraR (Battaglia et al., 2011), but whether these compounds are the actual inducers of these regulators has not been demonstrated. Previous studies indicated that accumulation of l-arabitol results in upregulation of genes encoding l-arabinose-releasing enzymes (de Vries et al., 1994), but this does not exclude the possibility that another intermediate of the pathway may be the actual inducer. It was shown in the d-galacturonic pathway from *A. niger* that the actual inducer of the pathway-related regulator is a down-stream intermediate of the pathway, 2-keto-3-deoxy-l-galactonate (Alazi et al., 2017).

To investigate the inducers of AraR and XlnR from *A. niger* a comparative transcriptome study on all intermediates of the PCP as well as the chiral alternatives of d-xylose and l-arabinose was performed, to determine which of these result in induction of AraR and/or XlnR controlled genes. A previous study demonstrated that *A. niger* can also grow on l-xylose and d-arabinose (unpublished data) and therefore these chiral alternatives were included to determine whether chirality affects the induction of XlnR and AraR. This was combined with a growth profile of a reference strain and several metabolic mutants that are blocked at various points of the PCP. Different from previous studies focusing only on the induction by a few pentoses and polyols (xylose, arabinose, l-arabitol and xylitol) on small set of target genes (arabinanolytic, xylanolytic and pentose catabolic genes) (Battaglia et al., 2011; Ishikawa et al., 2018; van Peij et al., 1998b), the integration of transcriptomes on all pathway intermediates, mutants, growth profiles and bioinformatics analysis in this study clarified the inducers of AraR and XlnR, providing a more comprehensive understanding of the complex regulation of pentose utilization in *A. niger*.

## 2 Materials and methods

### 2.1 Strains, media and growth conditions

*A. niger* N402 (Bos et al., 1988), CBS 138,852 (*cspA1, kusA*::*amdS, pyrA6*) (Alazi et al., 2016), CBS 144,530 (*cspA1, kusA*::*amdS, pyrA6*, Δ*larA*, Δ*xyrA*, Δ*xyrB*), CBS 144,672 (*cspA1, kusA*::*amdS, pyrA6*, Δ*ladA*, Δ*xdhA*, Δ*sdhA*) and CBS 144,042 (*cspA1, kusA*::*amdS, pyrA6*, Δ*xkiA*) (Chroumpi et al., 2021) were used in this study and were obtained from the Westerdijk Fungal Biodiversity Institute culture collection (Utrecht, The Netherlands). The strains were maintained by growth on *Aspergillus* Minimal Medium (MM) or Complete Medium (CM) (de Vries et al., 2004) plates (1.5 % w/v agar) at 30 °C with 1 % d-glucose and 1.22 g/L uridine (Sigma Aldrich). Growth profiles was performed using MM plates containing 1.5 % agar (w/v) with the addition of 25 mM monomeric substrates. All media were supplemented with 1.22 g/L uridine. Growth profile plates were inoculated with 1000 freshly harvested spores in 2 µL and incubated at 30 °C for up to 5 days. Growth was evaluated by visual inspection in triplicates.

### 2.2 Gene expression analysis

Previously published transcriptome data was used for cultures with, no carbon source (Lubbers et al., 2019), d-fructose, d-xylose and l-arabinose (Li et al., 2023), and the *araR* deletion mutant on l-arabinose (Chroumpi et al., 2022). For the other samples, freshly harvested spores from *A. niger* N402 were pre-grown in 250 ml CM containing 2 % d-fructose for 16 h at 30 °C in a rotary shaker at 250 rpm. After 16 h, mycelia were harvested by filtration through sterile cheesecloth, rinsed with MM, and approximately 2.5 g (wet weight) mycelium was transferred into 50 ml MM containing no carbon source or 25 mM of l-arabitol, xylitol, l-xylulose, d-xylulose, l-xylose or d-arabinose, in triplicate. Mycelia were collected after 2 h and were frozen in liquid nitrogen followed by storage at −80 °C. RNA isolation, purification, and quantitative and qualitative evaluation were performed as previously described (Garrigues et al., 2022).

Purification of mRNA, synthesis of cDNA library and sequencing were performed at the Joint Genome Institute (JGI, US). RNA sequencing, processing of raw reads were performed as previously reported (Garrigues et al., 2022).

DESeq2 v1.20 (Love et al., 2014) was used to determine differentially expressed genes (DEGs) between different growth conditions. Raw counts were used as DESeq2 input. The threshold of fold change (FC) ≥ 4 or ≤ 0.25 and adjusted P-value < 0.01 was used to define significant DEGs (higher-/lower- expressed genes). Heatmap and clustering were plotted using R package “ComplexHeatmap″ (Gu et al., 2016). The principal component analysis (PCA) was performed with the DEseq2 (Love et al., 2014), using the normalized counts by applying the *'regularized* log *(rlog)’* method. The genes showing significantly lower expression in AraR and XlnR mutants compared to corresponding reference strains grown on l-arabinose and d-xylose, and containing AraR binding (‘CGG[AGT]TAA[AT]’) (Ishikawa et al., 2018) or XlnR binding site (‘GGCTA[AG]’) (de Vries et al., 2002) in their promoter were chosen as regulon of AraR and XlnR, respectively. Based on the well-characterized function of AraR and XlnR in fungal plant biomass conversion (FPBC) (Battaglia et al., 2011), we further restricted the core regulons of XlnR and AraR to four major types of FPBC genes, including the sugar metabolic enzymes, the polysaccharide degrading carbohydrate-active enzymes (CAZymes), sugar transporters and transcriptional factors that were based on annotations from previous publications (Li et al., 2022, 2023; Peng et al., 2024; Xu et al., 2024).

## 3 Results and discussion

### 3.1 Deletion of PCP genes results in defined phenotypes

**Fig. 1:**
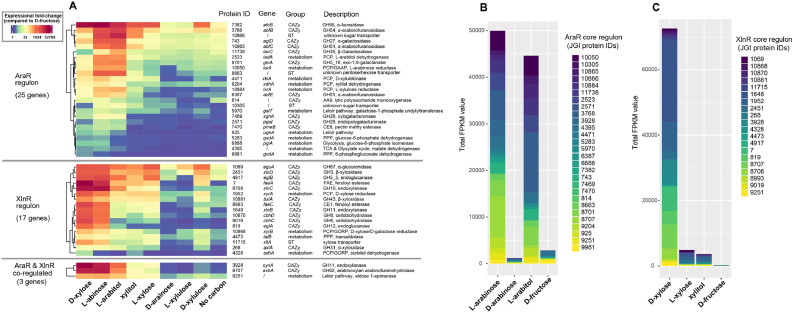
**Phenotypic analysis of the Pentose Catabolic Pathway (PCP) in *A. niger*.** A. Growth profile of *A. niger* strains on PCP intermediates and chiral alternatives on 5 cm petri dishes with MM and the carbon sources as indicated. As no variation was observed between duplicate plates, only one representative plate was imaged. B. Model for the PCP, indicating the enzymes involved in each step. The compounds in yellow boxes are not traditionally considered to be part of this pathway, but the growth profile suggest they are.

Previously, strains were constructed in which genes encoding three pentose reductases (*larA, xyrA, xyrB*), three polyol dehydrogenases (*ladA, xdhA, sdhA*) or d-xylulokinase were deleted (Chroumpi et al., 2021) and their phenotype was tested on d-xylose and l-arabinose. In this study the growth profile was expanded to include all PCP intermediates, as well as the chiral alternatives l-xylose, d-arabinose and d-arabitol (Fig. 1A). The reference strain was able to grow on all PCP intermediates as well as to a small extent on the chiral alternatives. The triple reductase mutant did not grow on d-xylose or l-arabinose, but was able to grow on the xylitol and l-arabitol as reported previously (Chroumpi et al., 2021). It also showed reduced growth on l- and d-xylulose, even though these genes are not involved in their conversion. Growth on l-xylose was reduced, suggesting a possible role for one of these genes in its conversion, while growth was unaffected on d-arabinose and d-arabitol. The triple dehydrogenase mutant showed no growth on all PCP intermediates upstream of xylitol, likely due to its inability to convert this polyol. It also showed no growth on l-xylose, while growth on d-arabinose and d-arabitol was unaffected in this mutant, demonstrating that l-xylose is also converted into xylitol by *A. niger*. The *xkiA* mutant showed no or strongly reduced growth on all carbon sources, likely due to it being blocked in the final step of the PCP. The residual growth on some substrates suggests that accumulation of these intermediates may result in the induction of alternative pathways.

Based on this growth profile, a modified map for the PCP of *A. niger* is proposed (Fig. 1B), in which l-xylose can also be reduced to xylitol, possibly by LarA, as this enzyme was shown to be active on l-xylose (Mueller et al., 2023). The KEGG database (Kanehisa et al., 2025) suggest a possible pathway of d-arabinose through d-arabitol to d-xylulose, although the genes involved have not been established for many species. Our data supports such a pathway considering the reduced growth of the *xkiA* mutant, but not the other mutants on d-arabinose and d-arabitol. Growth of the triple reductase mutant indicated that LarA, XyrA and XyrB do not play a major role in d-arabinose conversion, despite the previously reported activity of LarA on this substrate (Mueller et al., 2023). As growth of the triple dehydrogenase mutant on d-arabitol is similar to the reference strain it can also be concluded that LadA, XdhA and SdhA do not play a major role in the conversion of d-arabitol in *A. niger*. A d-arabitol dehydrogenase was identified in the plant pathogenic fungus *Uromyces fabae* (Link et al., 2005), which also showed activity with d-xylulose, suggesting it may be responsible for this conversion. The closest homolog of this gene in *A. niger* is NRRL3_1929, which belongs to the same PFAM family as LadA, XdhA and SdhA, and will be evaluated for this function in future studies.

The growth profile on the PCP intermediates and chiral alternatives not only confirmed the corresponding metabolic pathways (e.g., the conversion of l-xylose to xylitol, but also indicates complex regulation underlying the PCP. For instance, the poor growth of the reference strain on d-xylulose, l-xylulose and chiral alternatives compared to d-xylose and l-arabinose is likely mainly due to the absence of the inducers of the pathway genes (L-arabinose and xylitol) as these are upstream of these compounds. In addition, a lower efficiency of transmembrane transport for these compounds may affect this.

### 3.2 Establishing the XlnR and AraR regulon in A. niger

Previous studies revealed a large number of genes under control of XlnR and or AraR (Battaglia et al., 2011, 2014; de Souza et al., 2013; Gruben et al., 2017), as well as compensatory regulation of these two transcriptional activators (Battaglia et al., 2011; de Groot et al., 2003). The significant similarity in the binding site of these regulators (Ishikawa et al., 2018) and the presence of a small amount of l-arabinose in commercial d-xylose and of d-xylose in commercial l-arabinose (de Vries et al., 1998), complicates the determination of the inducer of these regulators. Therefore, in this study the regulon of XlnR and AraR in *A. niger* was re-evaluated by comparing the expression of a reference strain and deletion strains for AraR or XlnR. In total, 800 and 56 genes were significantly down regulated in Δ*araR* on l-arabinose and Δ*xlnR* on d-xylose compared to the reference strain, respectively (Table S1). Further analysis of the presence of AraR binding motif (‘CGG[AGT]TAA[AT]’) and XlnR binding motif (‘GGCTA[AG]’) in promoters of these two sets of genes identified 103 and 34 genes contain corresponding motifs, and they were defined as AraR and XlnR regulon (Fig. S1), respectively. Four genes were present in both regulons, likely due to the high similarity of the promoter binding sites (Ishikawa et al., 2018). This was previously reported for *axhA* (Battaglia et al., 2011; Gruben et al., 2017), where it was explained by the function of AxhA in releasing l-arabinose (relevant for AraR) from xylan (relevant for XlnR). Based on the well-characterized function of AraR and XlnR in fungal plant biomass conversion (FPBC), the core regulons of AraR and XlnR were further restricted to major FPBC genes (e.g., sugar metabolic enzymes, polysaccharide degrading CAZymes and sugar transporters), which identified 28 and 20 core regulon genes for AraR (Table 1) and XlnR (Table 2), respectively.

**Table 1:** **The AraR core regulon of *A. niger*.** The last column indicates the polysaccharide the enzyme acts on (CAZy) or the pathway it is involved in (metabolism). *P* = phosphate, PCP = pentose catabolic pathway, PPP = pentose phosphate pathway, GAAP = *d*-galacturonic acid pathway, TCA = tricarboxylic acid. Genes in bold are part of both the AraR and XlnR regulon.

Protein id	Gene name	Gene group	Function	Polysaccharide/Pathway
814		CAZy	AA9 lytic polysaccharide monooxygenase	Cellulose/xylan
7470	*pmeB*	CAZy	CE8 pectin methyl esterase	Pectin
8701		CAZy	GH5 exo-β−1,6-galactanase	Galactan/pectin
**3928**	***xlnA***	**CAZy**	**GH11 endoxylanase**	**Xylan**
743	*aglD*	CAZy	GH27 α-galactosidase	Galactomannan
2571	*pgaI*	CAZy	GH28 endopolygalacturonase	Pectin
7469	*xghA*	CAZy	GH28 xylogalacturonase	Pectin
11,738	*lacC*	CAZy	GH35 β-galactosidase	Pectin, xyloglucan
6387		CAZy	GH51 α-arabinofuranosidase	Pectin, xylan, xyloglucan
10,865	*abfC*	CAZy	GH51 α-arabinofuranosidase	Pectin, xylan, xyloglucan
3768	*abfB*	CAZy	GH54 α-arabinofuranosidase	Pectin, xylan xyloglucan
**8708**	***axhA***	**CAZy**	**GH62 α-arabinofuranosidase**	**Pectin, xylan, xyloglucan**
7382		CAZy	GH95 α-fucosidase	Xyloglucan, pectin
2523	*ladA*	Metabolism	L-arabitol dehydrogenase	PCP
10,050	*larA*	Metabolism	L-arabinose reductase	PCP, GAAP
4471	*xkiA*	Metabolism	D-xylulokinase	PCP
9204	*xdhA*	Metabolism	xylitol dehydrogenase	PCP
10,884	*lxrA*	Metabolism	L-xylulose reductase	PCP
5970	*galT*	Metabolism	galactose-1P uridyltransferase	Leloir pathway
925	*ugeA*	Metabolism	UDP-glucose-4-epimerase	Leloir pathway
**9251**		**Metabolism**	**aldose-1-epimerase**	**Leloir pathway**
5283	*gsdA*	Metabolism	glucose-6P-1-dehydrogenase	PPP
9981	*gndA*	Metabolism	6P-gluconate dehydrogenase	PPP
6888	*pgiA*	Metabolism	glucose-6P isomerase	Glycolysis
4395		Metabolism	malate dehydrogenase	TCA/glyoxylate cycle
10,866		Sugar transport	unknown	
8663		Sugar transport	pentose/hexose transporter	
10,305		Sugar transport	unknown

**Table 2:** **The XlnR core regulon of *A. niger*.** The last column indicates the polysaccharide the enzyme acts on (CAZy) or the pathway it is involved in (metabolism). PCP = pentose catabolic pathway, PPP = pentose phosphate pathway, GORP = *d*-galactose oxidoreductive pathway. Genes in bold are part of both the AraR and XlnR regulon.

Protein id	Gene name	Gene group	Function	Polysaccharide/Pathway
8993	*faeC*	CAZy	CE1 feruloyl esterase	Xylan, pectin
2451	*xlnD*	CAZy	GH3 β-xylosidase	Xylan
4917	*eglB*	CAZy	GH5 endoglucanase	Cellulose
10,870		CAZy	GH6 cellobiohydrolase	Cellulose
9019		CAZy	GH6 cellobiohydrolase	Cellulose
8708	*xlnC*	CAZy	GH10 endoxylanase	Xylan
**3928**	***xlnA***	**CAZy**	**GH11 endoxylanase**	**Xylan**
1648	*xlnB*	CAZy	GH11 endoxylanase	Xylan
819	*eglA*	CAZy	GH12 endoglucanase	Cellulose
268	*axlA*	CAZy	GH31 α-xylosidase	Xyloglucan
10,881		CAZy	GH43 β-xylosidase	Xylan
**8708**	***axhA***	**CAZy**	**GH62 α-arabinofuranosidase**	**Pectin, xylan, xyloglucan**
1069	*aguA*	CAZy	GH67 α-glucuronidase	Xylan
7	*faeA*		feruloyl esterase	Xylan, pectin
1952	*xyrA*	Metabolism	D-xylose reductase	PCP
10,868	*xyrB*	Metabolism	D-xylose/galactose reductase	PCP, GORP
4328	*sdhA*	Metabolism	sorbitol/xylitol dehydrogenase	PCP, GORP
4473	*talB*	Metabolism	transaldolase	PPP
**9251**		**Metabolism**	**aldose-1-epimerase**	**Leloir pathway**
11,715	*xltA*	Sugar transport	D-xylose transporter	

### 3.3 Identification of the monomeric inducer of XlnR and AraR in A. niger

Transcriptome analysis was performed on the *A. niger* reference strain grown on all PCP intermediates, as well as the chiral alternatives l-xylose and d-arabinose at 2 h after transfer of pre-grown mycelium to the specific carbon sources. This time point was chosen as previous studies (e.g., Battaglia et al., 2011, 2014; Gruben et al., 2017) revealed that at this time the mycelium has recovered from the transfer itself but has not yet consumed most of the substrate.

PCA analysis on these samples (Fig. S2) demonstrated the high similarity between the biological replicates and demonstrated the relative similarity between the conditions. The d-fructose samples are most distinct from the other samples, likely due to d-fructose being converted through glycolysis. d-Xylose and d-arabinose plot near each other, as do l-arabitol and xylitol. The largest cluster is formed by l- and d-xylulose, l-xylose, d-arabinose and no carbon source, which all resulted in poor growth of *A. niger* (Fig. 1). This may be due low induction of the corresponding catabolic genes due to absence of the real inducers. *A. niger* may therefore not induce the PCP during growth on these compounds. In addition, import of these carbon sources may be inefficient, as l- and d-xylulose do not occur naturally outside the cell, while l-xylose and d-arabinose occur very rarely in nature. *A. niger* may therefore lack efficient transporters for these sugars. Future studies should address the transporters and metabolic enzymes involved in utilization of these sugars in more detail to better explain the reduced growth that was observed.

**Fig. 2:**
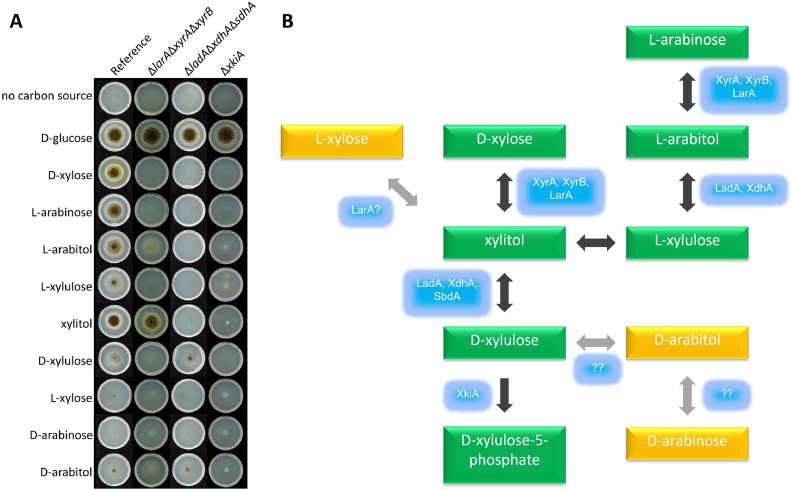
**Expression analysis of the core regulon of XlnR and AraR in *A. niger*.** A. Heatmap of the expression of the genes on PCP intermediates and no carbon source. B. Comparison of the summed-up expression levels of the AraR regulon on related carbon sources. C. Comparison of the summed-up expression levels of the XlnR regulon on related carbon sources.

Detailed analysis of the core regulon of XlnR and AraR revealed significant differences in expression during growth on the PCP intermediates (Fig. 2A). Genes of the AraR regulon were highly expressed on l-arabinose and l-arabitol, while expression was much lower on the other carbon sources. The next PCP intermediate, l-xylulose, does not result in high expression of the AraR regulon, indicating that the inducer of AraR is likely l-arabitol. This is supported by a previous study in the related fungus *Aspergillus nidulans* where a mutant with reduced l-arabitol dehydrogenase activity resulted in intracellular accumulation of l-arabitol and increased activity for l-arabinose reductase, l-xylulose reductase, xylitol dehydrogenase and α-arabinofuranosidase, all enzymes encoded by genes of the AraR regulon (de Vries et al., 1994). d-arabinose and d-arabitol do not result in high expression of the AraR regulon, not only supporting a pathway consisting of different enzymes but also demonstrating that the induction of AraR is chiral-sensitive. The similar induction pattern of l-arabinose and l-arabitol is also evident when the expression values of the core AraR regulon are summed up (Fig. 2B).

For the XlnR regulon, high expression was observed on d-xylose and l-arabinose (Fig. 2A). The latter can be explained by the contamination of commercial l-arabinose with some d-xylose (de Vries et al., 1998). It was previously shown that expression values of XlnR-regulated genes on d-xylose are the combination of induction by XlnR and repression by CreA, and that higher d-xylose concentrations result in lower expression values, as CreA appears to be sugar concentration dependent (de Vries et al., 1999). It is therefore not surprising that even the small amount of d-xylose in l-arabinose can lead to significant induction of the XlnR regulon. Expression on xylitol is strongly reduced for most XlnR-regulated genes, suggesting that d-xylose rather than xylitol is the XlnR inducer. The expression profile on l-xylose and xylitol are similar, likely due to the conversion of l-xylose through xylitol in the PCP and indicating that the induction of XlnR by d-xylose is chiral specific, similar to the induction of AraR by l-arabitol. As both d-xylose and l-xylose are converted into xylitol, but only d-xylose results in induction of XlnR target gene expression, this strongly supports that d-xylose and not xylitol is the XlnR inducer. The role of d-xylose as the inducer of XlnR is also evident when the expression values of the core XlnR regulon are summed up (Fig. 2C), as the total induced expression level is many times higher than on l-xylose or xylitol.

While we did not measure the metabolite levels in the samples used for transcriptomics, a previous study has shown that l-arabitol accumulated to higher level during growth of *A. niger* (both reference and AraR mutant) on l-arabinose than on d-glucose and d-xylose (de Groot et al., 2003). Another independent study also detected intracellular accumulation of l-arabitol and d-xylose during *A. niger* grown on l-arabinose and d-xylose, respectively, (Chroumpi et al., 2021; Witteveen et al., 1989). These findings support the induction of d-xylose and l-arabitol could occur intracellularly.

## 4 Conclusions

Identifying the inducers of transcriptional regulators is often complicated due to the contamination of commercial sugars with small amounts of other sugars and the reversibility of the steps of fungal sugar metabolism. In this study, a combination of growth profiling of metabolic mutants and transcriptome analysis of the wild type revealed the inducers for AraR (L-arabitol) and XlnR (D-xylose) in *A. niger*. In addition, the results demonstrated that the induction is chiral-specific as no induction of XlnR by l-xylose and AraR by d-arabitol was observed. Future studies could further refine the regulatory mode by addressing the influence of the inducer concentration, temporal patterns of gene expression using time course samples, and interaction with transporters and other regulators (e.g., CreA) to further decipher the complex regulation of fungal carbon metabolism. In addition, identification of the enzymes responsible for the conversion of l-xylose, d-arabinose and d-arabitol, as well as other pentoses also remains to be addressed.

## Funding sources

MP was supported by NWO ENW-XS program OCENW.XS23.4.051 to MP. The work (proposals 10.46936/10.25585/60001019 and 10.46936/10.25585/60001060) conducted by the U.S. Department of Energy Joint Genome Institute (https://ror.org/04xm1d337), a DOE Office of Science User Facility, were supported by the Office of Science of the U.S. Department of Energy Contract, no. DE-AC02–05CH11231. This work was supported in part by the U.S. Department of Agriculture, Agricultural Research Service.

## CRediT authorship contribution statement

**Mao Peng:** Data curation, Methodology, Formal analysis, Investigation, Writing – review & editing, Funding acquisition. **Wiebe Wennekers:** Data curation, Methodology, Formal analysis, Investigation. **Astrid Müller:** Methodology, Formal analysis, Investigation, Writing – review & editing. **Vivian Ng:** Project administration. **Anna Lipzen:** Data curation. **Igor V. Grigoriev:** Writing – review & editing, Funding acquisition. **Ronald P. de Vries:** Data curation, Supervision, Writing – original draft, Funding acquisition.

## Declaration of competing interest

The authors declare that they have no known competing financial interests or personal relationships that could have appeared to influence the work reported in this paper.

## Data Availability

The transcriptome data used in this study are available at the NCBI SRA database, with accession IDs: SRP448976, SRP449008-SRP449008, SRP449011-SRP449013, SRP449016, SRP449028, SRP449045, SRP449048, SRP449115, SRP449144-SRP449145, SRP449153-SRP449155, SRP449158, SRP449167, SRP606029, SRP606034, SRP606041, SRP606062, SRP606064, SRP606071.

## Appendix Supplementary materials

Image, application 1

Image, application 2

Image, application 3
