# Supplementary material for: Dissecting the complex regulation of pentose utilization in Aspergillus niger
Source: Curr Res Microb Sci. 2025 Sep 29;9:100482. doi: 10.1016/j.crmicr.2025.100482 (PMC12538316; doi:10.1016/j.crmicr.2025.100482)
Supplement: Supplementary file 1 [file mmc1.pdf]

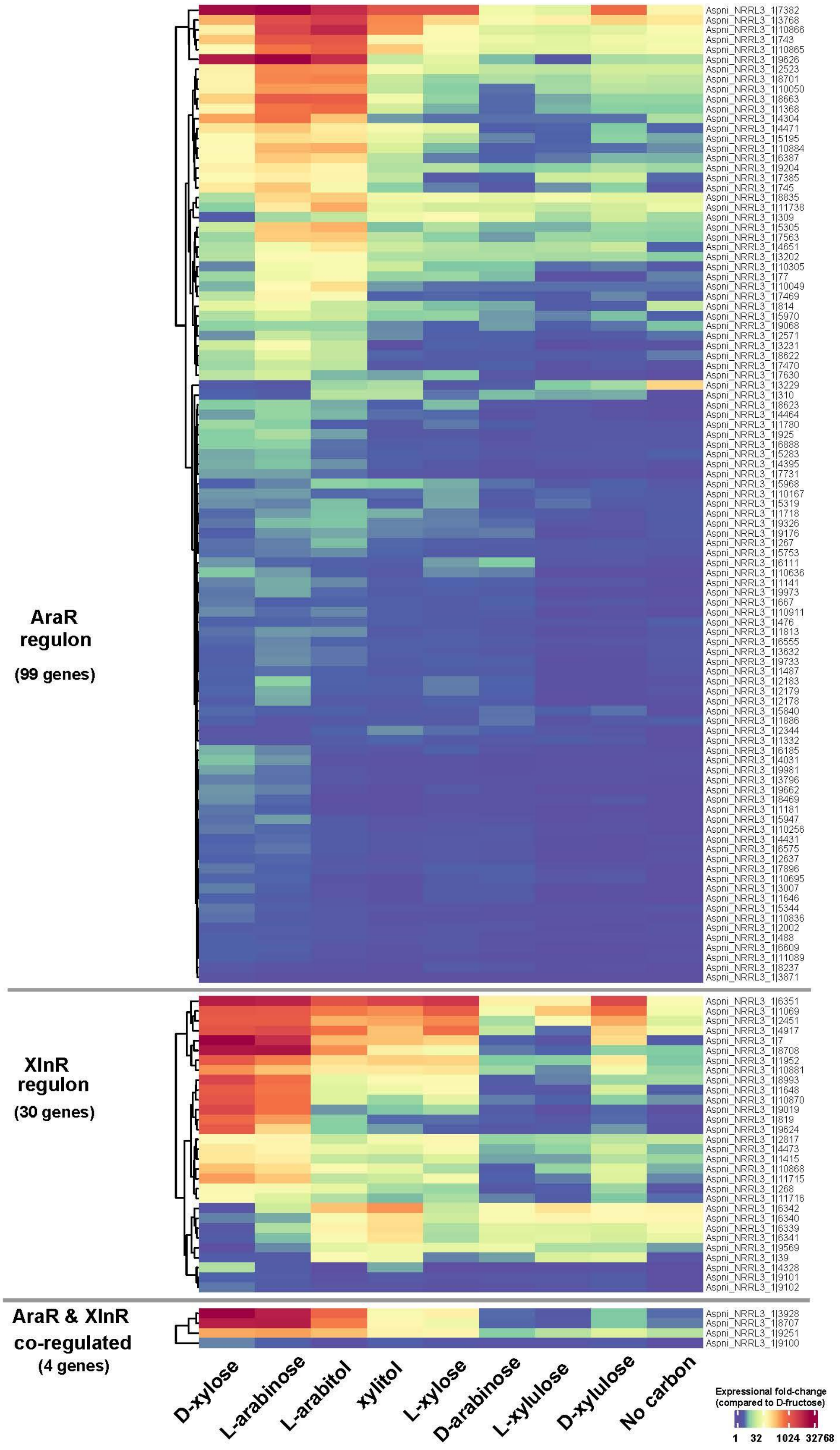

Figure S1. Heatmap of the expression of the genes belonging to the AraR and/or XlnR regulon. Values are the average on biological triplicate cultures on eight monosaccharides and a no carbon source control.
