# Supplementary material for: Dissecting the complex regulation of pentose utilization in Aspergillus niger
Source: Curr Res Microb Sci. 2025 Sep 29;9:100482. doi: 10.1016/j.crmicr.2025.100482 (PMC12538316; doi:10.1016/j.crmicr.2025.100482)
Supplement: Supplementary file 2 [file mmc2.pdf]

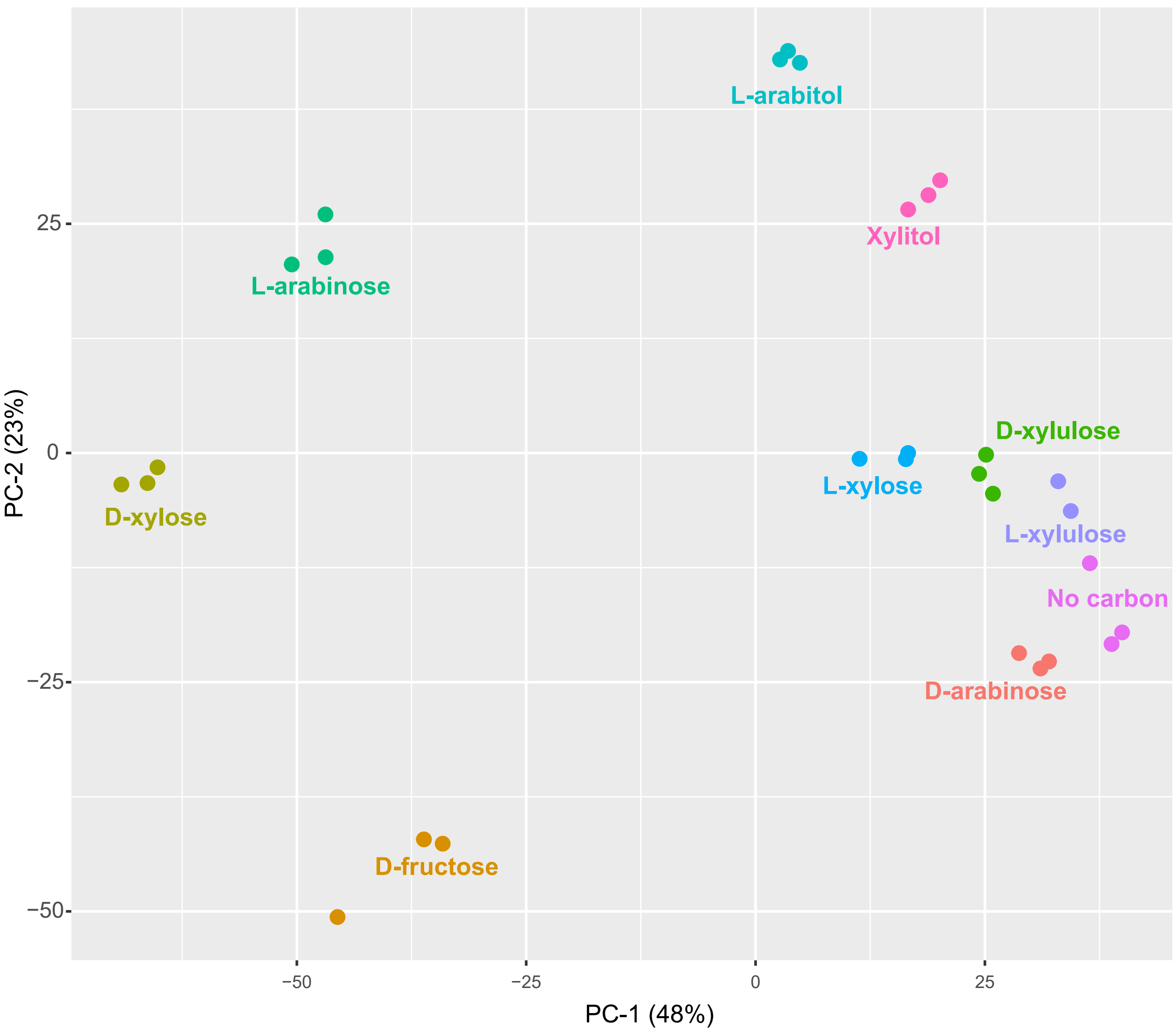

Figure S2. Principal component analysis of *A. niger* grown on 9 carbon sources and a no carbon source control. The percentage of variance explained with the principal components (PC-1 and PC-2) was shown in bracket. Three biological replicates of each condition were indicated with dots of the same color.
